# Monoamine Oxidase Inhibitory Constituents of Propolis: Kinetics and Mechanism of Inhibition of Recombinant Human MAO-A and MAO-B

**DOI:** 10.3390/molecules191118936

**Published:** 2014-11-18

**Authors:** Narayan D. Chaurasiya, Mohamed A. Ibrahim, Ilias Muhammad, Larry A. Walker, Babu L. Tekwani

**Affiliations:** 1 National Center for Natural Products Research, Research Institute of Pharmaceutical Sciences, School of Pharmacy, University of Mississippi, University, MS 38677, USA; E-Mails: ndchaura@olemis.edu (N.D.C.); mmibrahi@olemiss.edu (M.A.I.); milias@olemiss.edu (I.M.); lwalker@olemiss.edu (L.A.W.); 2 Departments of BioMolecular Sciences, School of Pharmacy, University of Mississippi, University, MS 38677, USA

**Keywords:** monoamine oxidase A, monoamine oxidase B, galangin, apigenin, propolis, flavonoids

## Abstract

Propolis is the resinous material that bees gather from leaf buds, flowers and vegetables. Propolis extracts contain constituents with a broad spectra of pharmacological properties and are important ingredients of popular dietary supplements. Propolis extracts were evaluated *in vitro* for inhibition of recombinant human monoamine oxidase (MAO)-A and MAO-B. The dichloromethane extract of propolis showed potent inhibition of human MAO-A and MAO-B. Further fractionation identified the most active fractions as rich in flavonoids. Galangin and apigenin were identified as the principal MAO-inhibitory constituents. Inhibition of MAO-A by galangin was about 36 times more selective than MAO-B, while apigenin selectivity for MAO-A *vs.* MAO-B was about 1.7 fold. Apigenin inhibited MAO-B significantly more potently than galangin. Galangin and apigenin were further evaluated for kinetic characteristics and the mechanism for the enzymes’ inhibition. Binding of galangin and apigenin with MAO-A and -B was not time-dependent and was reversible, as suggested by enzyme-inhibitor binding and dissociation-dialysis assay. The inhibition kinetics studies suggested that galangin and apigenin inhibited MAO-A and -B by a competitive mechanism. Presence of prominent MAO inhibitory constituents in propolis products suggests their potential for eliciting pharmacological effects that might be useful in depression or other neurological disorders. The results may also have important implications in drug-dietary supplement interactions.

## 1. Introduction

Propolis is the resinous material that bees gather from leaf buds, flowers and certain vegetables [1]. The bees gather this and transform it, in order to disinfect the beehive, seal cracks, build panels, as well as using it as an antimicrobial agent and disinfectant. The types of propolis are distinctive in different places because of variability in regional plant varieties. However, the principal characteristics and constituents of propolis remain significantly similar. The importance of propolis is due to the great number of active ingredients present [2]. Its alcoholic extract (called tincture) is well known and used for therapeutic purposes, principally for its stimulant action on the organism’s defense system. It is also used as antioxidant and anti-microbial agent, stimulant, and for its burn healing and anti-inflammatory activity [3,4,5]. Propolis is highly rich in polyphenols and flavonoid constituents, which make propolis preparation important constituents of dietary supplements and also suggest their potential therapeutic use in cardioprotective, vasoprotective, antioxidant, antiatherosclerotic, anti-inflammatory and antiangiogenic applications [6]. Propolis extracts were evaluated *in vitro* for inhibitions of recombinant human monoamine oxidase (MAO) -A and B. The studies were further extended to identify the principal MAO inhibitory constituents in the propolis extracts.

MAO-A and MAO-B (EC.1.4.3.4) are FAD-dependent enzymes responsible for the metabolism of neurotransmitters such as dopamine, serotonin, adrenaline, and noradrenaline and for the inactivation of exogenous arylalkyl amines [7,8]. Both enzymes are bound to the outer mitochondrial membrane and catalyze the oxidative deamination of their substrates. Although they share 70% sequence identity, MAO-A and B exhibit different substrate and inhibitor specificities; serotonin and norepinephrine are preferentially metabolized by MAO-A and phenylethylamine, benzylamine, dopamine by MAO-B, whereas clorgyline and l-deprenyl selectively inhibit MAO-A and B, respectively. Due to their central role in neurotransmitters metabolism, these enzymes represent attractive drug targets in the pharmacological therapy of neurodegenerative diseases and depression [9,10,11,12]. In particular, MAOs appear to form the first line of defense against monoamines absorbed from foods, such as tyramine and 3-phenylethanolamine, which would otherwise produce an indirect sympathomimetic response resulting in the precipitous rise in blood pressure known as the “cheese effect” [13]. Identification of MAO inhibitors is of great interest in drug discovery [14]. Recent efforts toward the development of MAO inhibitors are focused on selective MAO-A or MAO-B inhibitors. Selective MAO-A inhibitors are effective in the treatment of depression [15], whereas MAO-B inhibitors are useful for the treatment of depression, Alzheimer’s disease and Parkinson’s disease [11,16]. Evaluation of natural products resources, botanicals and other dietary supplements for MAO inhibitory constituents is of great interests, due to possible use of dietary supplements in improving neurological disorders as well as their possible interactions with drugs and the food rich in dietary-monoamines [17,18]. Herbal natural products have been suggested as important source for inhibitors of MAOs and also support traditional use of these herbal products as alternative for treatment of depression, Parkinson’s disease and other neuropsychiatric as well as neurological disorders [19] Specially, the dietary supplements and herbal preparations containing β-carboline harmala alkaloids show prominent inhibition of MAO-A and have been suggested to be responsible for their psychoactive properties [20,21,22,23].

The present studies have identified two flavones, namely galangin and apigenin, as principal MAO inhibitory constituents in propolis extracts. We also report the kinetic characteristics of inhibition of MAO-A and B by galangin and apigenin and the properties of their binding with the recombinant enzymes. These studies may have possible implications in use of propolis-based dietary supplements and standardized propolis extracts in the improvement of neurological disorders, which are associated with disfunctions in pathways involved in the degradation, synthesis or transport of biogenic monoamines.

## 2. Results and Discussion

### 2.1. Determination of Inhibitory Effect of Galangin and Apigenin on MAO-A and -B

**Figure 1:**
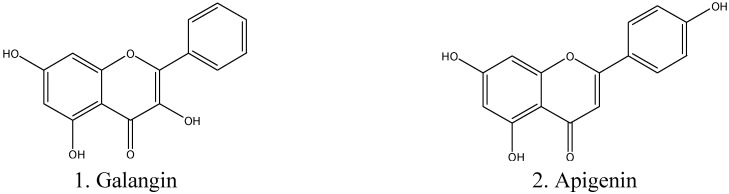
Structure of galangin (**1**) and apigenin (**2**).

The dichloromethane (DCM) extract of propolis (Prop-E) was evaluated *in vitro* against recombinant human MAO-A and -B, whereby the Prop-E extract demonstrated potent MAO-A and B inhibitory activities (Table 1). The inhibition of MAO-A by Prop-E was about 10-fold more potent (IC_50_ 0.60 μM) compared to the inhibition of MAO-B (IC_50_ 6.99 μM). DCM extract of Prop-E was subjected to preparative HPLC fractionation and the fractions were evaluated *in vitro* against recombinant human MAO-A and B. The preparative fractions #1 and #2 were identified as highly active fractions, with almost equal inhibition of MAO-A and -B, while fraction #3 showed more potent inhibition of MAO-B than A (Table 1). The most active fraction #2 was found to contain several flavonoids (Supplementary Data Figures S1 and S2). Based on the calculated peak areas percentage of individual flavonoids in the fraction were as follows: taxifolin ≥ 0.1%, morin ≥ 0.65%, quercetin ≥ 1.18%, fisetin ≥ 2.4%, apigenin ≥ 0.3%, and galangin ≥ 12.9%. Galangin and apigenin were identified as the most prominent MAO inhibitory constituents (Table 1 and Figure 1).

**Table 1:** Inhibition of recombinant human Monoamine Amine Oxidase-A and B by propolis extract, fractions and pure constituents.

Sample Name	Unit	Monoamine Oxidase-A (IC_50_) *	Monoamine Oxidase-B (IC_50_) *
Propolis Extract (prop-E)	μg/mL	0.60 ± 0.12	6.99 ± 0.09
Fraction 2 (9.0–17.5 min) ^#^	μg/mL	0.33 ± 0.03	0.31 ± 0.02
Fraction 3 (17.5–20 min) ^#^	μg/mL	0.16 ± 0.11	0.23 ± 0.09
Fraction 4 (20–30 min) ^#^	μg/mL	5.36 ± 0.22	0.98 ± 0.08
Fraction 5 (30–35 min) ^#^	μg/mL	26.16 ± 0.29	22.19 ± 3.34
Galangin	μM	0.13 ± 0.01	3.65 ± 0.150
Apigenin	μM	0.64 ± 0.11	1.12 ± 0.27
Quercetin	μM	2.44 ± 0.12	38.66 ± 1.20
Fisetin hydrate	μM	2.10 ± 0.09	>100
Morin hydrate	μM	5.88 ± 0.15	49.66 ± 0.56
Toxifolin	μM	65.06 ± 1.96	53.80 ± 3.91
Clorgyline	μM	0.0065 ± 0.0003	-
Deprenyl	μM	-	0.036 ± 0.0012
Harmine	μM	0.0039 ± 0.0003	27.50 ± 2.67

* The IC_50_ values computed from the dose response inhibition curves are Mean ± S.D. of triplicate observations. ^#^ *Retention time* (*Tr* values, indicate time for start and end for collection of the fraction. See Figure S1, Supplementary Data.

Both galangin and apigenin were more potent and selective inhibitors of MAO-A than B, with 28- and 1.7-fold selectivity, respectively towards MAO-A (Table 2). The additional flavonoids identified in the active propolis fractions, namely quercetin, fisetin and morin, were also more active against MAO-A (*i.e.*, 15-, >47- and 9-fold, respectively) than MAO-B. However, the inhibition of MAO-A and B by these flavonoids was significantly less potent compared to galangin and apigenin (Table 1). Therefore, apigenin and galangin were investigated in more details for kinetics and mechanism of inhibition of MAO-A and MAO-B.

**Table 2:** Inhibition/binding affinity constants (Ki) values for inhibition of recombinant human MAO-A and B by galangin and apigenin.

Compound	Monoamine Oxidase-A		Monoamine Oxidase-B	
	Ki (μM) *	Type of Inhibition	Ki (μM) *	Type of Inhibition
Galangin	0.029 ± 0.004	Competitive/Reversible	1.998 ± 0.039	Competitive/Reversible
Apigenin	0.125 ± 0.014	Competitive/Reversible	0.238 ± 0.024	Competitive/Reversible
Harmine	0.0019 ± 0.0002	Competitive/Reversible	-	-
Clorgyline	0.0026 ± 0.006	Mixed/Irreversible	-	-
Deprenyl	-	-	0.0301 ± 0.003	Mixed/Irreversible

* Values are mean ± S.D. of triplicate experiments.

### 2.2. Evaluation of Inhibition Mechanism and Kinetics

**Figure 2:**
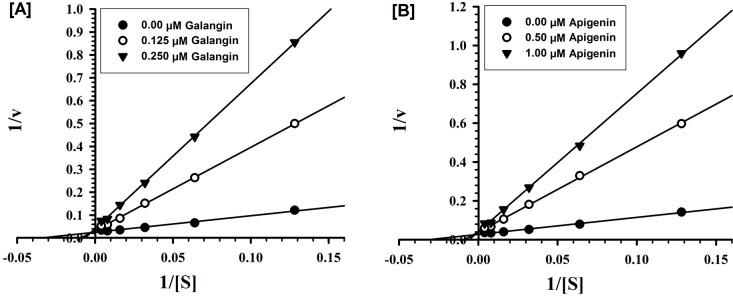
Kinetic characteristics of inhibition of recombinant human MAO-A with [**A**] galangin and [**B**] apigenin; V = nmoles/min/mg protein and S = substrate kynuramine concentration (μM).

**Figure 3:**
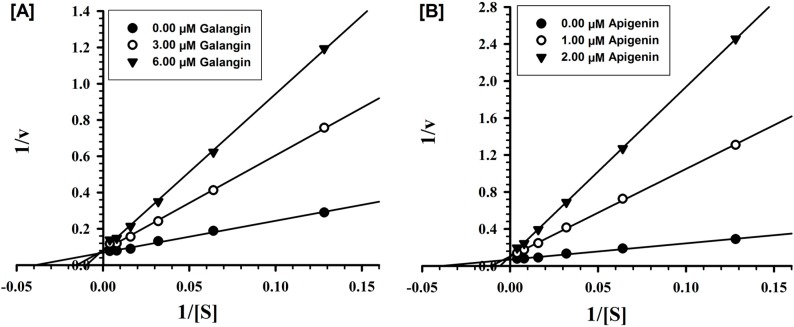
Kinetic characteristics of inhibition of recombinant human MAO-B with [**A**] galangin and [**B**] apigenin; V = nmoles/min/mg protein and S = substrate kynuramine concentration (μM).

Detailed *in vitro* studies were carried out to understand the kinetics and mechanism of inhibition of recombinant human MAO-A and -B by galangin and apigenin. Both galangin and apigenin were tested against MAO-A and -B at varying concentrations of kynuramine, a nonselective substrate, to investigate the nature of inhibition of the enzymes by these constituents. Based on dose-response inhibition, two concentrations of the inhibitors were selected, one below and another above IC_50_ value for the inhibition. For each experiment, three sets of assays were done at varying concentrations of the substrate, one control without inhibitors and with two concentrations of the inhibitor. The enzyme kinetics data are presented as double reciprocal Lineweaver-Burk plots (Figure 2 and Figure 3). The Ki (inhibition constant) values and other enzyme kinetics parameters were computed with SigmaPlot 12.3 enzyme module. Binding of galangin and apigenin with human MAO-A increase the K_M_ value (the Michaelis-Menten constant) with no apparent effect on the V_max_ (maximum enzyme activity), indicating that the inhibition of MAO-A by these constituents was competitive, and substrate-inhibitor binding with the common enzyme active site (Figure 2).

Similarly, the inhibition of MAO-B by galangin and apigenin was also competitive (Figure 3). Ki values computed by SigmaPlot enzyme module are presented in Table 2. The affinities for binding determined as inhibition constants (Ki) of galangin and apigenin with MAO-A and -B were compared with the standard inhibitors clorgyline and deprenyl respectively. Galangin exhibited significantly higher affinity of binding for MAO-A (Ki-0.029 μM) than for B (1.998 μM). While affinity of binding of apigenin was only marginally higher for MAO-A (Ki-0.123 μM), compared to the MAO-B (Ki-0.238 μM).

### 2.3. Analysis of Time-Dependent Enzyme Inhibition

To analyze if the binding of galangin and apigenin with MAO-A and B to produce enzyme inhibition was time-dependent, the enzymes were pre-incubated for different time periods (0–15 min) with the inhibitor at the concentrations, which produce approximately 60%–80% inhibition of the enzyme.

**Figure 4:**
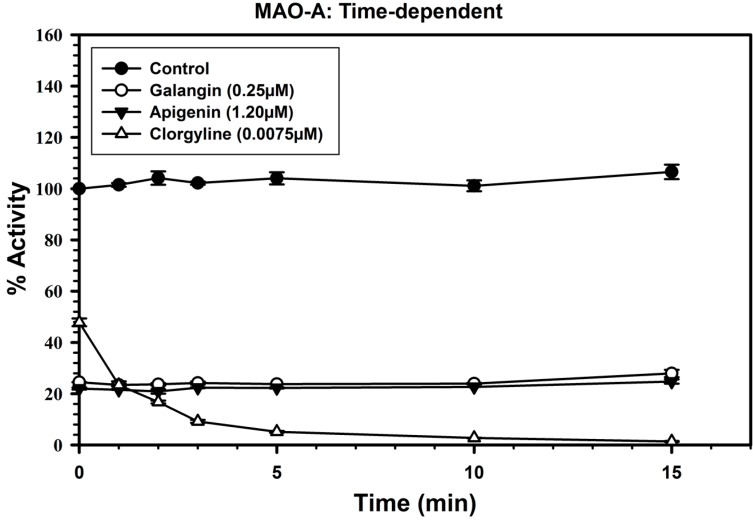
Time-dependent inhibition of recombinant human MAO-A by galangin (0.25 μM), apigenin (1.2 μM) and clorgyline (7.5 nM). The remaining activity was expressed as % of activity. Each point represents mean ± S.D. of triplicate values.

**Figure 5:**
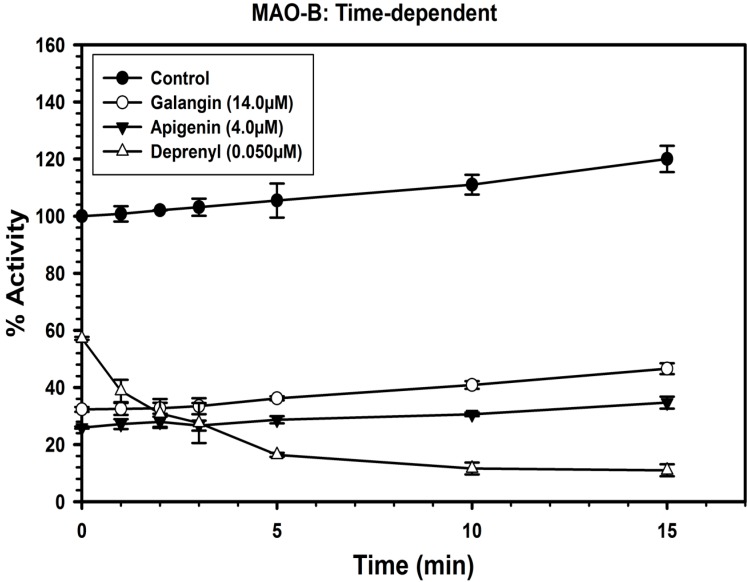
Time-dependent inhibition of recombinant human MAO-B by galangin (14.0 μM), apigenin (4.0 μM) and l-deprenyl (0.050 μM). The remaining activity was expressed as % of activity. Each point represents mean ± S.D. of triplicate values.

The inhibitor concentrations used to determine the time-dependent enzyme inhibitions were, galangin (0.25 μM), apigenin (1.2 μM) and clorgyline (7.5 nM) for MAO-A (Figure 4), while galangin (14.0 μM), apigenin (4.0 μM) and deprenyl (50 nM) for MAO-B (Figure 5).

The controls without inhibitors were also run simultaneously. Activities of the enzymes were determined as described above and percentage of enzyme activity remaining was plotted against the preincubation time to determine time-dependent inhibition. The binding/inhibition of MAO-A and B with galangin and apigenin was not dependent on pre-incubation time (Figure 4 and Figure 5).

### 2.4. Analysis of Binding of Galangin and Apigenin with MAO-A and -B

**Figure 6:**
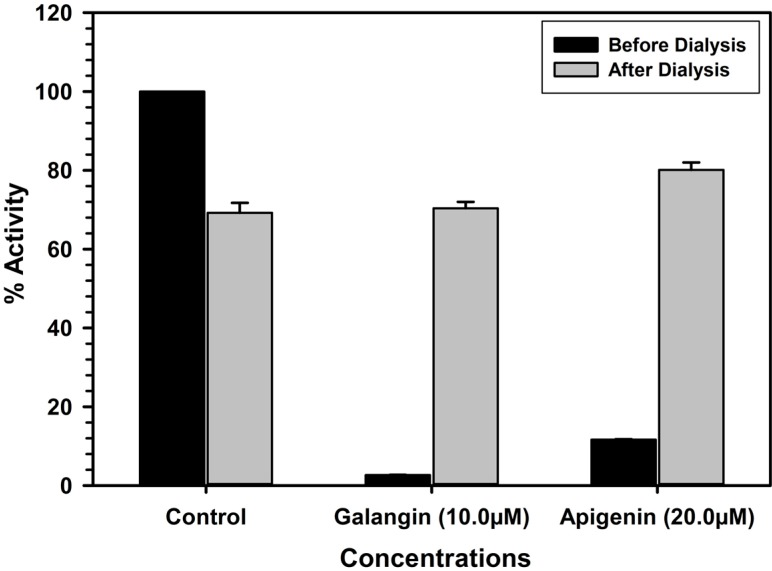
Analysis of nature of binding of galangin and apigenin with recombinant human MAO- A by recovery of catalytic activity of the enzyme after dialysis dissociation. Each bar shows mean ± S.D. of triplicate values.

The characteristic binding of these inhibitors was also investigated by the enzyme-inhibitor complex dissociation by dialysis. High concentrations of the inhibitors were allowed to interact with the enzyme (MAO-A or B) for 20 min and the resulting enzyme-inhibitor complex mixtures were dialyzed overnight against buffer solutions. The catalytic activities of the enzymes were analyzed before and after the dialysis. The recombinant human MAO-A lost about 30% of the enzyme activity during overnight dialysis. Incubation of MAO-A with 10.0 μM of galangin or 20.0 μM apigenin caused more than 97% and 88% inhibition of the catalytic activity of the enzyme respectively. After the dialysis about 70% and 80% activity of the enzyme was recovered from enzyme-galangin and enzyme-apigenin incubation mixtures. This observation indicated reversal of the enzyme activity due to formation of dissociable enzyme-inhibitor complex. Thus, the binding of galangin and apigenin with MAO-A was reversible. The recombinant MAO-B lost about 30% of the enzyme activity during overnight dialysis. Incubation of galangin (50.0 μM) with MAO-B produced almost 58.3 inhibition of the enzyme activity respectively, which was almost completely recovered after dialysis (Figure 6).

**Figure 7:**
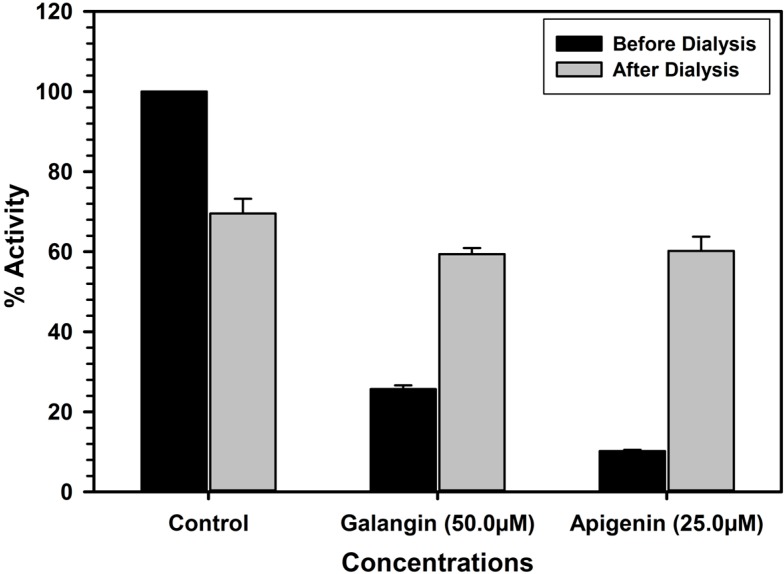
Analysis of nature of binding of galangin and apigenin with recombinant human MAO- B by recovery of catalytic activity of the enzyme after dialysis dissociation. Each bar shows mean ± S.D. of triplicate values.

Similarly Incubation of MAO-B with apigenin (25.0 μM) inhibited about 90% catalytic activity of the enzyme, which was almost fully recovered after dialysis of the enzyme-apigenin incubation mixture (Figure 7).

### 2.5. Discussion

The results presented in this paper demonstrate inhibition of MAO-A and MAO-B as an important pharmacological action of the propolis extracts. A broad spectrum of pharmacological properties, such as anticancer/antitumor, antimicrobial, immunomodulatory, antioxidant and neuroprotective activities, has been exhibited by propolis extracts and preparations [3,4,5]. The broad spectrum of pharmacological and biological properties in propolis preparations may be attributed to the more than 300 natural identified constituents [6,24,25]. The prominent groups of natural products identified in propolis are polyphenols, phenolic aldehydes, coumarins, amino acids and steroids [1,2,25]. The predominant MAO-A inhibitory property of propolis extracts and fractions indicates their possible effect in elevating the levels of biogenic monoamines. A recent study has also reported inhibition of crude MAO activity in rat liver mitochondrial preparations [26]. These results suggest that use of propolis preparations along with the diet rich in dietary monoamines (most prominently tyramine) may conceivably increase levels of the dietary monoamines in circulation. The protein containing foods have naturally low levels of tyramine, but aging and storage of these foods, for the purpose of developing good taste, results in an increase in levels of tyramine in these foods such as aged cheeses, cured meats, fermented cabbage, soya sauce, fish sauce, yeast extract spreads and broad bean pods. Consumption of these foods is contraindicated with use of irreversible MAO inhibitors (MOAI) particularly for the treatment of depressive disorders [27,28].

The original propolis extract showed 10 fold more selectivity for inhibition of MAO-A than MAO-B Further, bioassay guided fractionation identified a fraction rich in flavonoids. Among these flavonoids identified in the active propolis fraction 2, galangin and apigenin were found to be the prominent MAO inhibitory constituents of propolis. Identity of apigenin and galangin in active propolis fraction (fraction #2) was further confirmed with HRESIMS. Previous studies have also reported apigenin and galangin in propolis [29,30,31]. Both galangin and apigenin were significantly less potent against MAO-B as compared to MAO-A. The 30-fold selectivity of galangin for MAO-A and its presence at 13% in propolis extracts indicates that galangin may contribute to selective inhibition of MAO-A by propolis extracts. MAO binding of these constituents with MAO-A and B was competitive with the non-specific substrate (kynuramine), time-independent and reversible. Galangin and apigenin are naturally occurring flavones; commonly present in several plants and fruits. Earlier studies have also demonstrated MAO inhibitory action of flavonoids [32]. However, this is the first report on inhibition of MAO-A and -B with galangin. Galangin is commonly used as a food additive and has been shown to have broad spectrum of pharmacological activities such as induction of apoptosis and autophagy in HepG2 cells [33], protection against ischemic injury and potential neuroprotective activity [34], anti-inflammatory properties via negative regulation of NF-κB [35], alleviation of liver damage through inhibition of liver fibrosis [36], attenuation of mast cell mediated allergic inflammation [37], anti-obesity effect through inhibition of pancreatic lipases [38], prevention of osteoclastic bone destruction and osteoclastogenesis [39] and antiparasitic/antibacterial activities. Similarly, potential biological effects reported earlier with apigenin include antioxidative, anti-inflammatory and anticancer and cancer prevention activities [40]. The significant MAO inhibitory properties of galangin and apigenin as reported herein suggest additional potential utility of these two flavones for alleviation of neurological disorders associated with depletion of monoamines, the most important of which are depression and Parkinson’s disease. The reversible and selective inhibition of MAO-A by propolis extracts and galangin may be important for their potential use in treatment resistant depression (TRD), implicated in 33%–57% cases of depression [41]. TRD is currently treated by electroconvulsive therapy (ECT) or augmentation of serotonin selective reuptake inhibitors (SSRIs) with antipsychotics. Use of ECT and antipsychotics are associated with safety and tolerability concerns. Depression is hypothesized to result from the deregulation and depletion of monoamine neurotransmitters possibly due to hyperactive MAO-A. Reversible monoamine oxidase-A inhibitors (RIMAs) have shown efficacy in treatment of depression, with better safety and tolerability compared to SSRIs [41,42]. With regard to the MAO inhibitory property of galangin and apigenin reported in this study, previous studies have also reported MAO inhibitory activities of a few other flavonoids [43,44,45]. However, the results reported earlier are highly variable due to different enzyme sources and preparations employed in the different studies. Most of these studies were conducted with crude mitochondrial preparations from rat brain, which may not be suitable for determination of selective effect of the test compound on specific MAO isoforms. Apigenin has been shown to inhibit total MAO (A & B) in mouse brain crude mitochondrial preparation with IC_50_ of 1 μM. Further, inhibition of MAO-A by apigenin was about 7.5-fold more potent than of MAO-B in these preparations as determined by use of iso-enzyme specific enzyme substrates. The results presented here clearly indicate selectivity of active propolis fractions and the compounds identified from the active fractions.

Potent MAO-A and MAO-B inhibitory property of galangin and apigenin may be important for application of propolis products/extracts for management of neurological disorders, which are associated with depletion of biogenic monoamines. However, issues related to bioavailability of flavonoids, specially limited bioavailability to the brain, have to addressed for their use in treatment or improvements of neurological disorders. Apigenin has also been shown to have a unique monoamine transporter activator activity [46] and stimulate adult neurogenesis *in vivo* and *in vitro*, by promoting neuronal differentiation [47]. This study has also shown that apigenin promoted learning and memory performance in the Morris water task. Based on the previous reports, together with the results reported herein supports the potential application of propolis dietary supplements, consisting of two potent MOAI active flavones galangin and apigenin, in the management of neurological disorders. The results reported in this study suggest the development of propolis preparation standardized for their apigenin and galangin constituents as potential treatments for depression and other neurological disorders.

## 3. Experimental Section

### 3.1. Materials and Reagents

Human recombinant monoamine oxidase A and monoamine oxidase B were purchased from BD Biosciences (Bedford, MA, USA). Kynuramine, clorgyline, deprenyl and DMSO were obtained from Sigma Chemical (St Louis, MO, USA). Propolis extract powder (NCNPR # 15902 as Prop-E) analyzed in this study was procured commercially. The propolis used for preparation of the extract powered was originally sourced from China. Samples used in this work were preserved using the standard procedures for procurement, processing and packaging at the NCNPR. The reference standards of six flavonoids, namely galangin, apigenin, fisetin, quercetin, morin, and taxifolin were purchased from Sigma–Aldrich (St. Louis, MO, USA). The identities of the reference standards have been confirmed by comparing their ^1^H- and ^13^C-NMR spectroscopic data, using a Brucker 400 MHz instrument, with those reported in the literature. The UHPLC/APCI-MS was carried on Agilent 1290 UHPLC system coupled with an Agilent 6120 single quadrupole mass spectrometer. The high-resolution mass spectra (HRMS) were obtained using a Bruker BioApex spectrometer.

### 3.2. Preparation of Propolis Extracts

A sample of propolis extract powder (10 g) was macerated with dichloromethane (DCM, 100 mL) at room temperature and kept overnight. Then the extracts were sonicated and filtered using Whatman^®^ filter paper (90 mm Ø), and were ried using a Savant Speed Vac Plus SC210A Concentrator. These extracts along with the fractions as mentioned below were submitted for MAO-A and -B inhibition assays.

### 3.3. Fractionation of Propolis Extract and Analysis of MAO Inhibitory Constituents by HPLC

The HPLC system consisting of a Waters DeltaPrep 4000 series HPLC System equipped with a 2487 dual absorbance detector and a computerized data station equipped with the Waters Empower 2 software (Waters, Milford, MA, USA) was used for the preparative fractionation of the propolis. Separation was achieved on a C_18_ 100 Å column (Phenomenex, 250 mm × 21.20 mm, I.D.; 10 μm particle size; Phenomenex Inc., Torrance, CA, USA). The HPLC system consisted of Waters 2795 Separations Module equipped with PDA Waters 996 detector and a computerized data station equipped with Waters Empower 2 software (Waters) was used for the analytical work. Separation was achieved on a C_18_ 100 Å column (Phenomenex, 150 mm × 4.6 mm, I.D.; 5 μm particle size).

For the fractionation of propolis DCM extract using preparative HPLC (C_18_ 100 Å column; Phenomenex, 250 mm × 21.20 mm), the mobile phase consisted of water (A) and acetonitrile (B), both containing 1.0% acetic acid which were applied in the following gradient elution; 90% A/10% B hold for 2 min; increased to 100% B in next 30 min and hold on 100% B for 5 min. The total run time was 37 min, and flow rate was 15 mL/min. Detection wavelength was at 254 and 280 nm. The injection volume was 500 μL. A 56 mg DCM Prop-E extract was fractionated by Waters DeltaPrep 4000 series HPLC to afford five main fractions; the first fraction 0–9 min (1.5 mg), second fraction 9–17.5 min (11.0 mg), third fraction 17.5–20 min (6.5 mg), fourth fraction 20–30 min (20 mg), and the fifth fraction 30–35 min (4.0 mg); (Figure S1; supporting Information). The fractions 2–4 were submitted for MAO inhibitory activities (Table 1).

For the identification of MAO inhibitory constituents using analytical HPLC (C_18_ 100 Å column; Phenomenex, 150 mm × 4.6 mm), the mobile phase consisted of water (A) and acetonitrile (B), both containing 1.0% acetic acid were applied in the following gradient elution: 90% A/10% B hold for 2 min; increased to 100% B in next 30 min and hold on 100% B for 5 min. The total run time was 37 min, and flow rate was 1.0 mL/min. The peaks were assigned based on their retention times (Figure 2; supporting Information). Detection wavelength was at 254 and 280 nm. The injection volume was 10 μL. The two most potent markers apigenin and galangin showed up at retention time (*Tr*) 14.5 min and 18.2 min, respectively. Calculated peak areas were used for relative quantification of individual flavonoids. Based on the calculated peak areas percentage of individual flavonoids in the propolis extract were as follows, taxifolin ≥ 0.1%, morin ≥ 0.65%, quercetin ≥ 1.18%, fisetin ≥ 2.4%, apigenin ≥ 0.3%, and galangin ≥ 12.9%.

For confirmation of the identities of both apigenin and galangin, UHPLC/APCI-MS analysis was performed followed by the isolation of the compounds of interest and running HRESIMS (Figures S3–S8, Supplemental data). UHPLC/APCI-MS analysis was performed on an Agilent 1290 Infinity liquid chromatograph (Agilent, Santa Clara, CA, USA) coupled with an Agilent 6120 single quadrupole mass spectrometer. The LC column was a Waters Acquity UPLC BEH RP-C18 column (1.7 μm, 2.1 × 150 mm). The mobile phase consisted of A (acetonitrile with 0.05% formic acid) and B (water with 0.05% formic acid) at a flow rate of 0.5 mL/min. The gradient elution started with 90% A and then it was increased linearly to 100% A in 20 min and held for 5 min. The column temperature was maintained at 30 °C. The compounds of interest were analyzed by ESI and APCI in both the positive and negative modes. The drying gas flow was 10 L/min, and the nebulizer pressure was 30 psi. The drying gas temperature and vaporizer temperature were set to 250 and 200 °C, respectively. The capillary voltage was 3000 V, and the corona current was 4.0 μA.

For HRESIMS, the mass detector was a time of flight (Model G1969A) equipped with an electrospray ionization interface and was controlled by Agilent software (Agilent MassHunter Work Station, A.02.01). The negative mode acquisition was performed with a capillary voltage of 3000 V, and nitrogen was used as nebulizer gas (30 psig) as well as drying gas at 10 L/min at drying gas temperature of 300 °C. The voltage of PMT, fragmentor and skimmer was set at 750 V, 125 V and 60 V respectively. Full scan mass spectra were acquired from *m*/*z* 100–1100. Data acquisition and processing was done using the MassHunter Workstation software (Qualitative Analysis Version B.05.00). HRESIMS for apigenin and galangin *m*/*z* 269.0442 [M−H]^−^ (calcd for C_15_H_9_O_5_, 269.04500).

### 3.4. MAO Inhibition Assays

To investigate the effect of the selected constituents on MAO-A and MAO-B, the kynuramine deamination assay was adapted for 96-well plates as described earlier [23,24]. A fixed substrate concentration and varying inhibitor concentrations were used to determine the IC_50_ value at the point where 50% inhibition of the catalytic activity of the enzyme occurred. For MAO-A, the substrate concentration of 80 μM kynuramine was chosen because the apparent K_M_ value for substrate binding reported previously was approximately 40 μM [48]. Since Km is the substrate concentration at half Vmax, therefore, 2 × K_M_ (2 × 40 = 80 μM), was selected for determining IC_50_ values. Similarly, for MAO-B, substrate concentration of 50 μM kynuramine was chosen. The assay was performed with the addition of inhibitor. Inhibition was calculated as percent of product formation compared to the corresponding control (enzyme-substrate reaction) without the inhibitors. The reactions were carried out in 0.1 M potassium phosphate buffer at pH 7.4. Incubations mixtures contained 5 μg/mL of MAO-A (50 μL in buffer) and 10 μg/mL of MAO-B (50 μL in buffer). The inhibitor was dissolved in DMSO or in buffer (if not dissolved in DMSO). The total reaction volume was 200 μL yielding a final DMSO concentration of 1.0% in the reaction mixture. The reaction mixtures were pre-incubated for 10 min at 37 °C followed by the addition of MAO-A/MAO-B to initiate the reactions. Reactions were incubated for 20 min at 37 °C and were stopped immediately by the addition of 75 μL of 2N NaOH. The formation of 4-hydroxyquinoline was determined fluorometrically by SpectraMax M5 fluorescence plate reader (Molecular Devices, Sunnyvale, CA, USA) with an excitation and emission wavelength of 320 nm and 380 nm, respectively, using the Soft Max Pro program. Appropriate controls with the test compounds, fractions and extracts, where the enzyme or the substrate was added after stopping the reaction were setup simultaneously to check interference with the fluorescence measurements. None of the test compounds, fractions and extracts showed any interference with fluorescence measurement.

### 3.5. Determination of IC_50_ Values

IC_50_ values for inhibition of MAO-A and B by the selected constituents were determined using fixed concentration of substrate and varying concentration of the inhibitor. Galangin (0.001 μM to 100 μM), apigenin (0.001 μM to 100 μM) and clorgyline (0.10 nM to 100 nM) for MAO-A and galangin (0.01 μM to 100 μM), apigenin (0.01 μM to 100 μM) and deprenyl (0.01 μM to 100 μM) for MAO-B were tested to determine the IC_50_, from the dose-response inhibition curves using XL-Fit^©^.

### 3.6. Enzyme Kinetics Studies

For determination of the enzyme inhibition constants (Ki) for inhibition of MAO-A and MAO-B with apigenin and galangin the enzyme assays were carried out at different concentration of kynuramine substrate (1.90 μM to 500 μM) and at least two fixed concentrations (one above and one below IC_50_ values) of the inhibitors/compounds [galangin (0.125 μM and 0.25 μM), apigenin (0.5 μM and 1.0 μM) for MAO-A and galangin (3.0 μM and 6.0 μM), apigenin (1.0 μM and 2.0 μM) for MAO-B] were used to determine the K_m_ and V_max_ values in presence of the inhibitor. Controls without inhibitor were also run simultaneously. The results are presented as double reciprocal Lineweaver-Burk plots and the kinetic data namely Km, Vmax and Ki values were computed by SigmaPlot 12.3 using Enzyme-Kinetics module employing Michaelis-Menten equation. The results were also analyzed with SigmaPlot 12.3 using Enzyme-Kinetics module to determine the type of inhibition.

### 3.7. Analysis of Time-Dependent Enzyme Inhibition

To analyze if the binding of the inhibitors with MAO-A and B, for producing the inhibition of catalytic function, was time-dependent the enzyme was pre-incubated for different time periods (0–15 min) with the inhibitor at the concentration, which produces approximately >60% inhibition. The inhibitor concentrations used to test time-dependent inhibition were galangin (0.25 μM), apigenin (1.2 μM) and clorgyline (7.5 nM) with MAO-A (20 μg/mL) and; galangin (14.0 μM), apigenin (4.0 μM) and deprenyl (50 nM) with MAO-B (50 μg/mL). The controls without inhibitors were also run simultaneously. Activities of the enzymes were determined as described above and percentage of enzyme activity remaining was plotted against the preincubation time to determine time-dependent enzyme-inhibition.

### 3.8. Analysis of Reversibility and Binding of the Inhibitor with MAO-A and B

Most of the inhibitors produce inhibition of the target enzyme through formation of an enzyme-inhibitor complex. Formation of the enzyme-inhibitor complex may be accelerated in presence of high concentration of the inhibitor. The reversibility of binding of MAO inhibitory galangin and apigenin were determined by formation of the complex by incubating the enzyme with high concentration of the inhibitor followed by extensive dialysis of the enzyme-inhibitor complex and recovery of catalytic activity of the enzymes. MAO-A (0.05 mg/mL protein) enzyme was incubated with the test compounds; galangin (10.0 μM) and apigenin (20.0 μM) and MAO-B (0.05 mg/mL protein) enzyme was incubated with compound; galangin (50.0 μM) and apigenin (25.0 μM) in a total reaction mixture volume of 1 mL, containing 100 mM potassium phosphate buffer (pH 7.4). After 20 min incubation at 37 °C, the reaction was stopped by chilling the tubes on the ice bath. All the samples were dialyzed against potassium phosphate buffer (25 mM; pH 7.4) at 4 °C for 14 h (three buffer changes). Control enzyme (without inhibitor) was also run through the same procedure and activity of the enzyme was determined before and after the dialysis. Recovery of the catalytic activity enzyme after extensive dialysis of the enzyme-inhibitor mix provided the information about reversibility/irreversibility of binding of the inhibitor with the enzyme.

## 4. Conclusions

Propolis, a natural wax-like resinous substance present in bee hives, has been extensively used in dietary supplements and also as folk medicine for treatment of several diseases, including neurological disorders. We have identified apigenin and galangin as prominent MAO-inhibitory constituents in propolis extracts. The presence of prominent monoamine oxidase inhibitory constituents in these products suggests their potential for eliciting pharmacological effects that might be useful in depression or other neurological disorders.

## Supplementary Materials

Supplementary materials can be accessed at: http://www.mdpi.com/1420-3049/19/11/18936/s1.

## Supplementary Files

Supplementary File 1
