# Supplementary material for: Monoamine Oxidase Inhibitory Constituents of Propolis: Kinetics and Mechanism of Inhibition of Recombinant Human MAO-A and MAO-B
Source: Molecules. 2014 Nov 18;19(11):18936–52. doi: 10.3390/molecules191118936 (PMC6271006; doi:10.3390/molecules191118936)

Supplementary

Figure S1. HPLC chromatogram of fractionation of dichloromethane extract of Propolis.

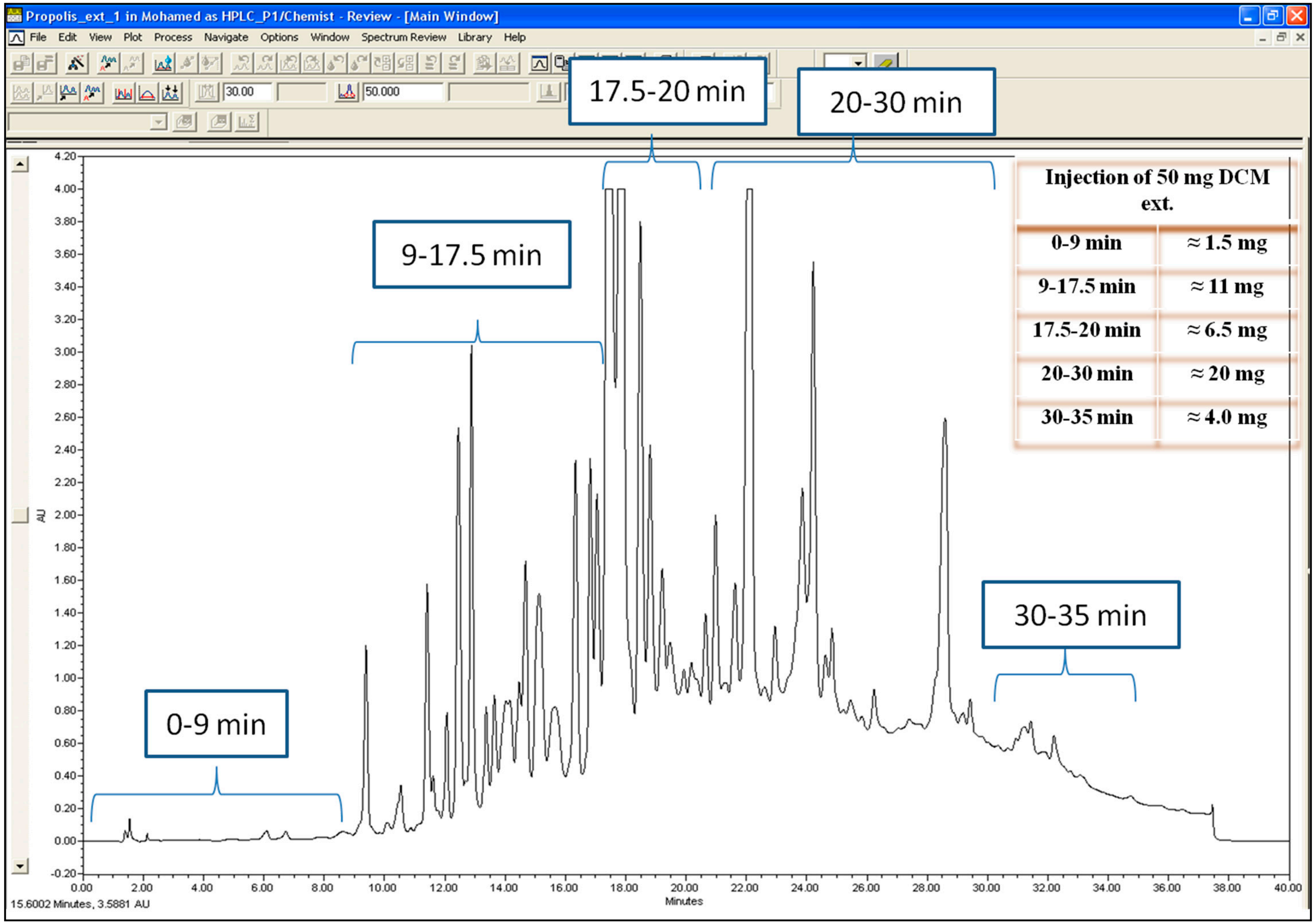

Figure S2. Identification of active constituents in Fraction 2 (15.5–20 min). (**A**) The HPLC chromatogram of the fractions, (**B**) The HPLC chromatogram of authentic reference standards. 1 (taxifolin, Tr 10 min), 2 (morin, Tr 12 min), 3 (quercetin, Tr 12.8 min), 4 (fisetin, Tr 13.5 min), 5 (apigenin, Tr 15.0 min), and 6 (galangin, Tr 18.0 min).

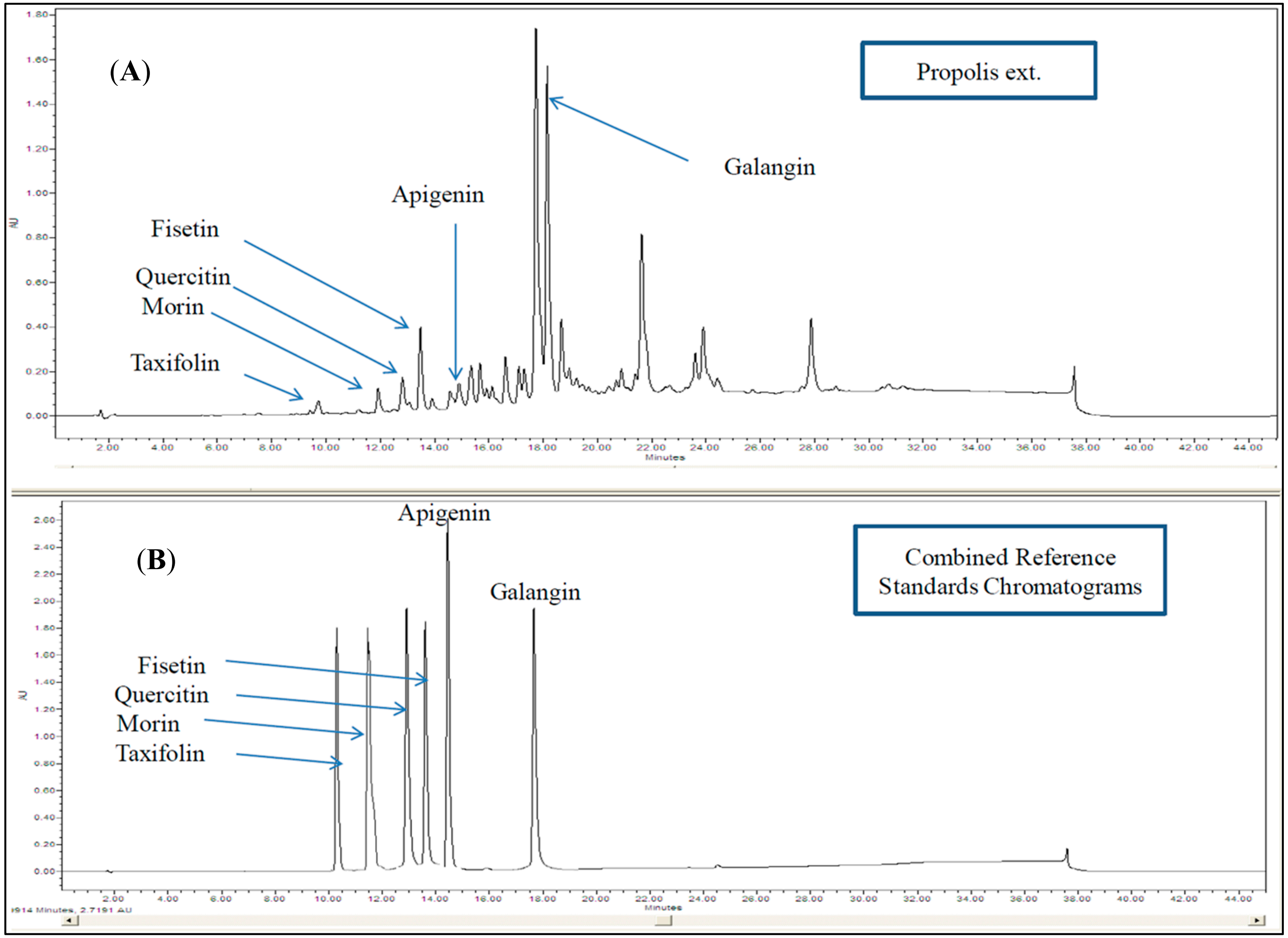

Figure S3. Individual HPLC chromatograms for Apigenin, Morin and Taxifolin.

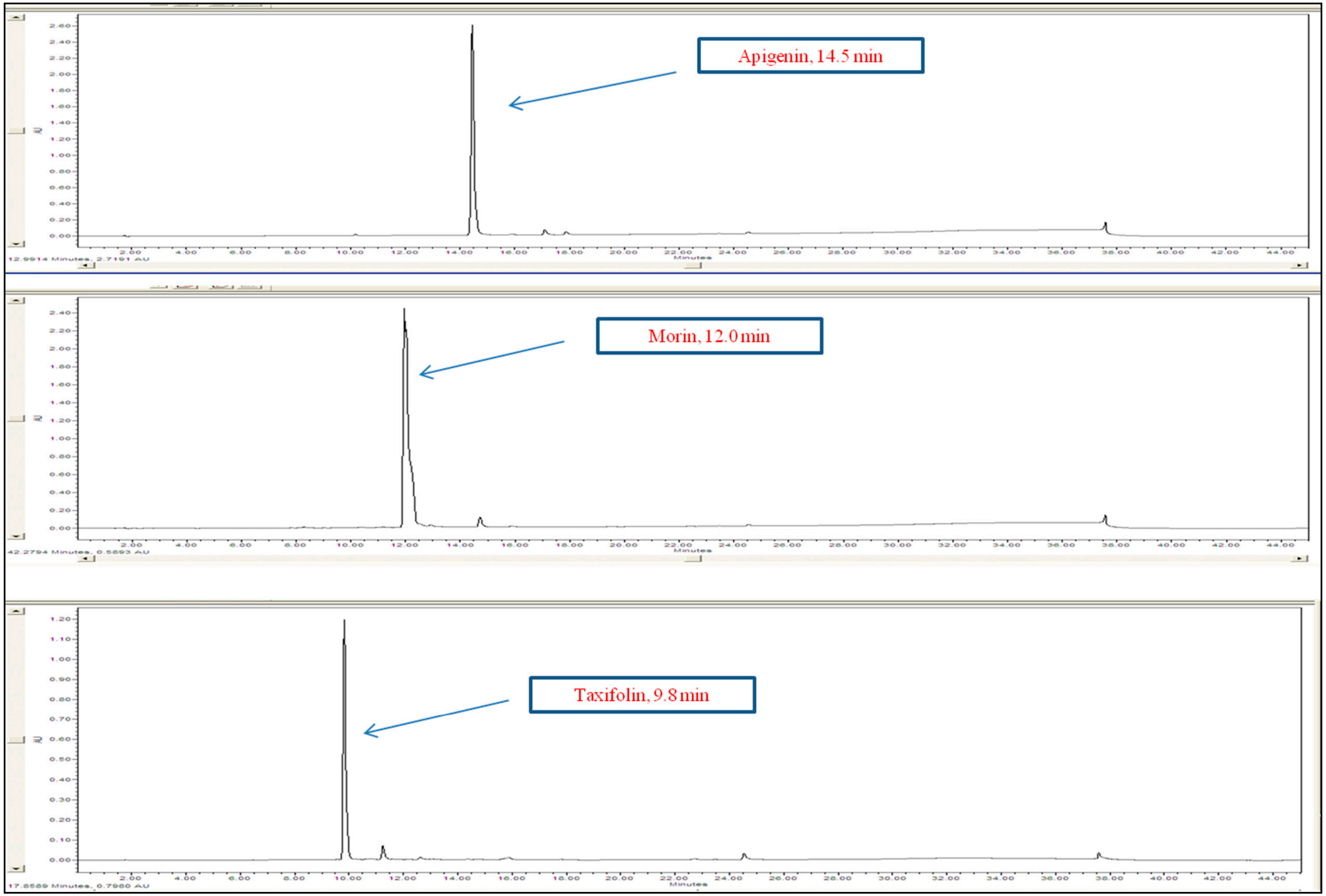

Figure S4. Individual HPLC Chromatograms for Quercetin and Galangin.

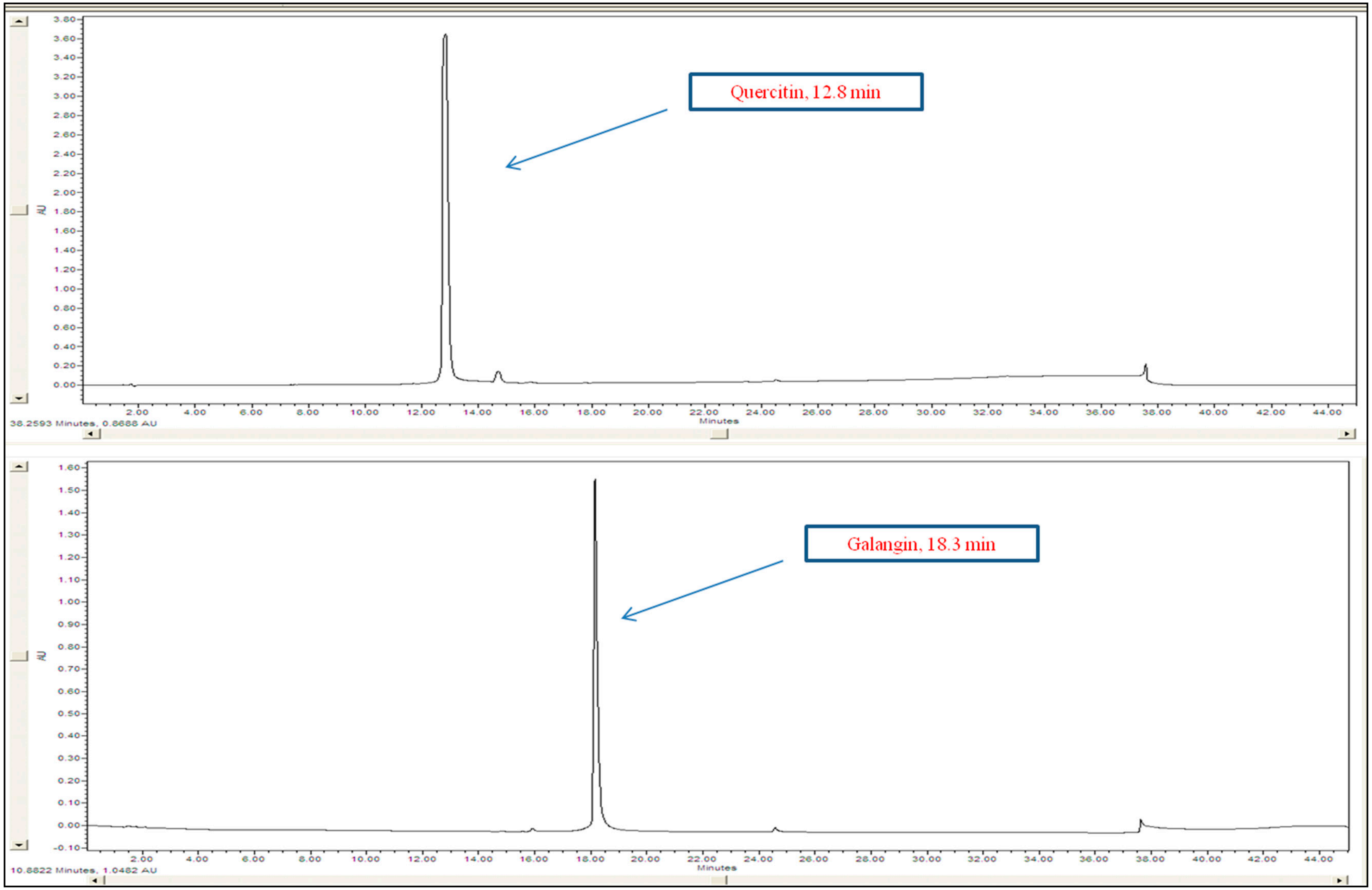

Figure S5. UHPLC/APCI-MS for the apigenin isolated from propolis.

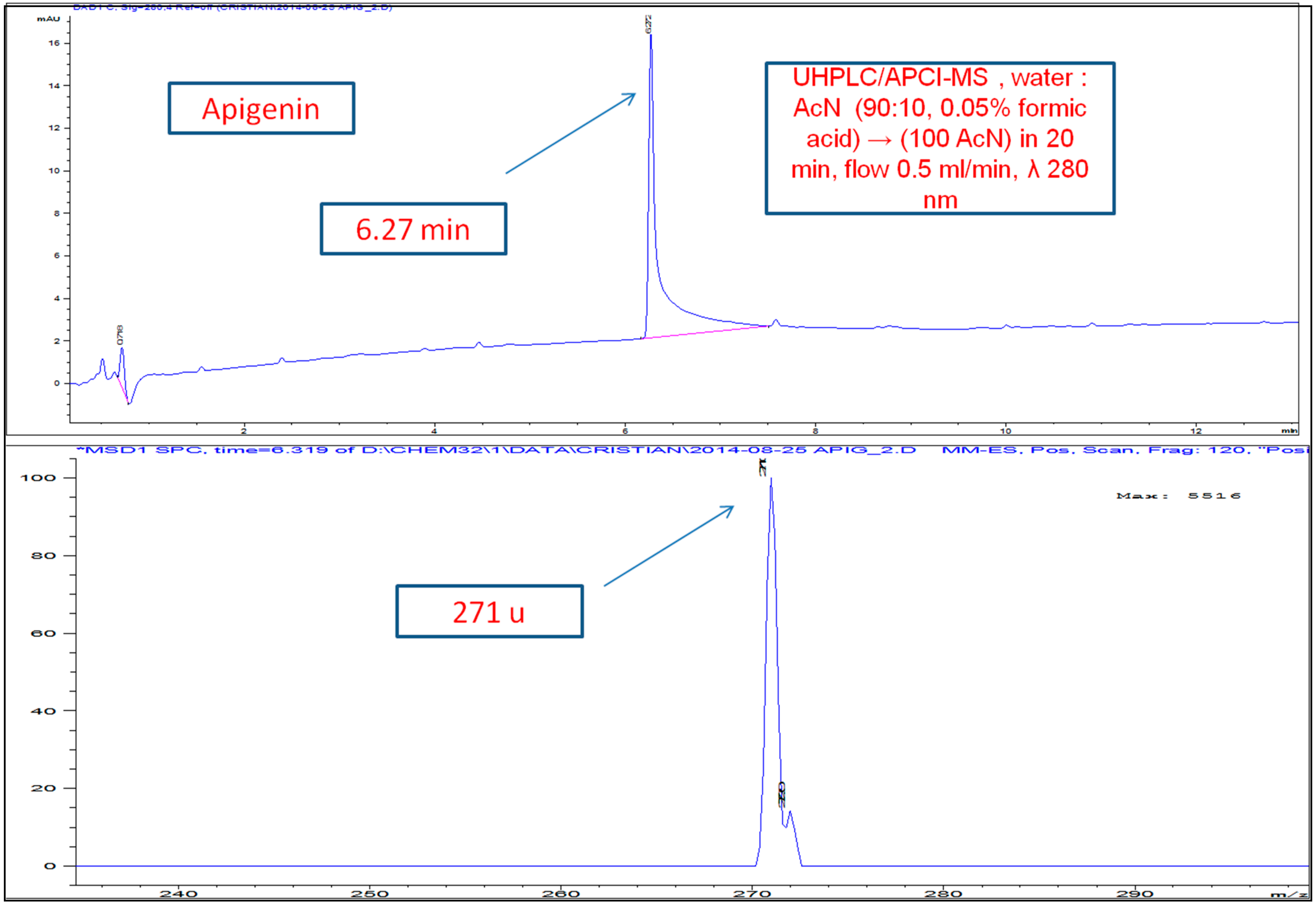

Figure S6. HRMS for the Apigenin isolated from propolis.

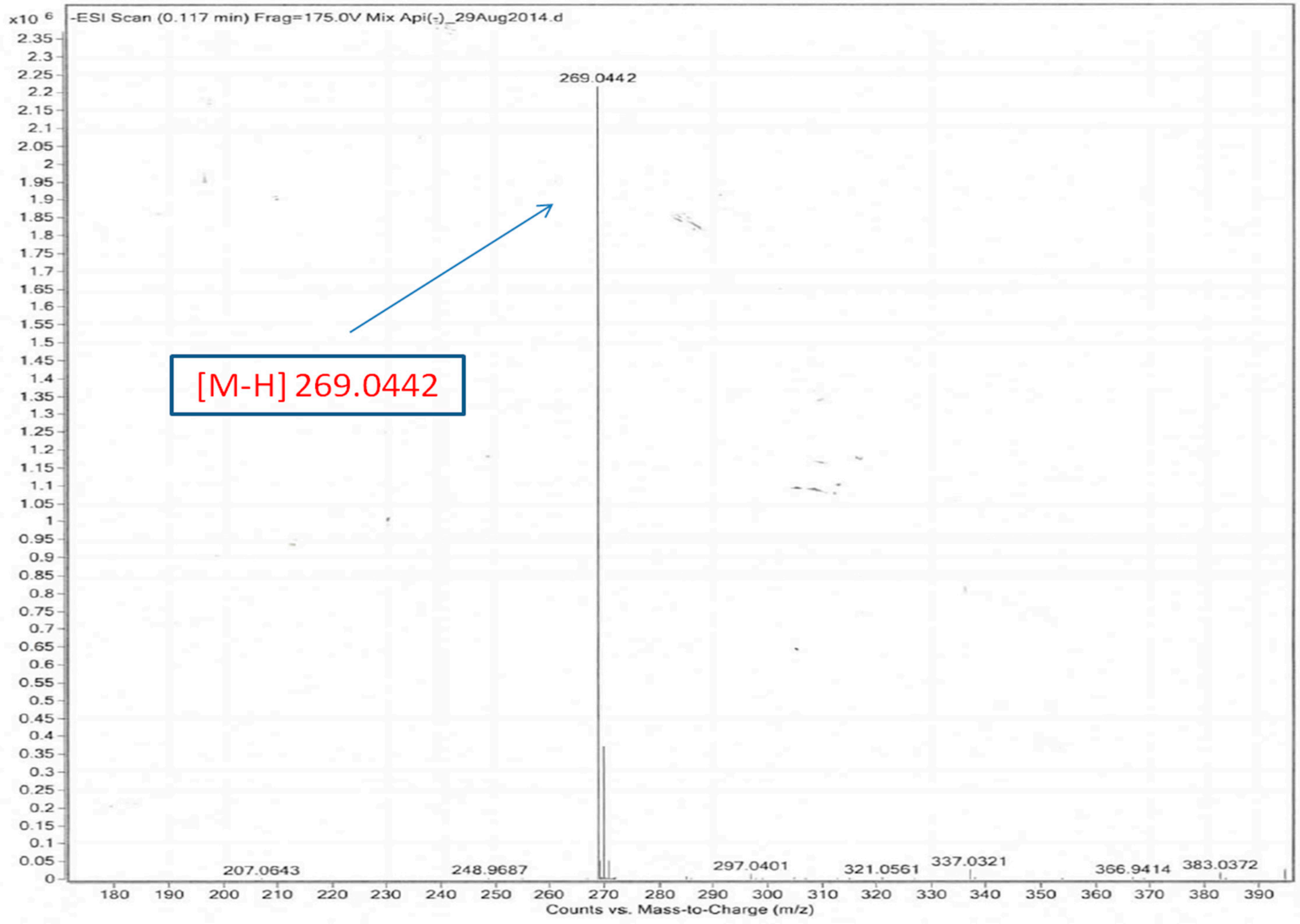

Figure S7. UHPLC/APCI-MS for the galangin isolated from propolis.

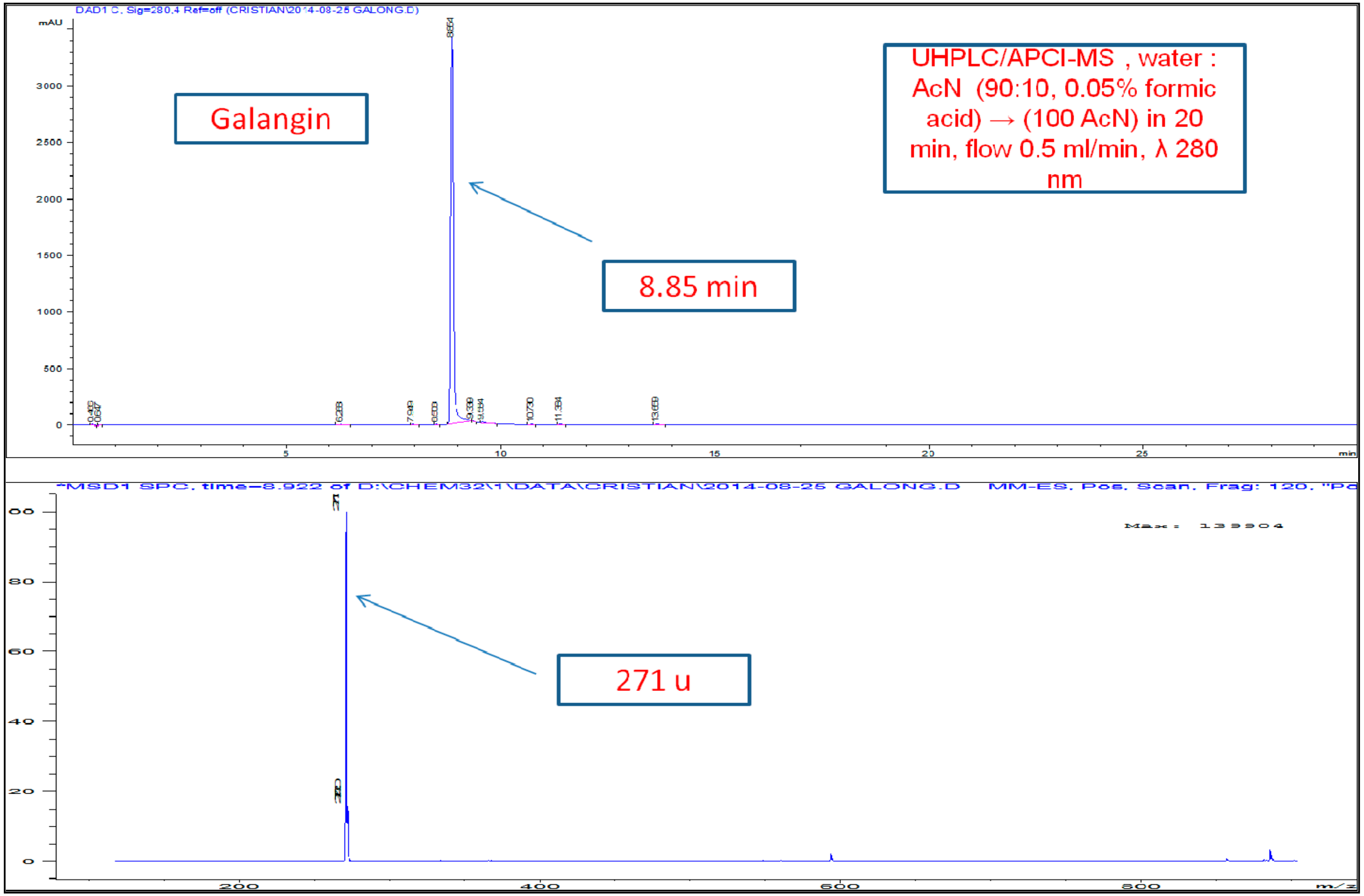

Figure S8. HRMS for galangin isolated from propolis.

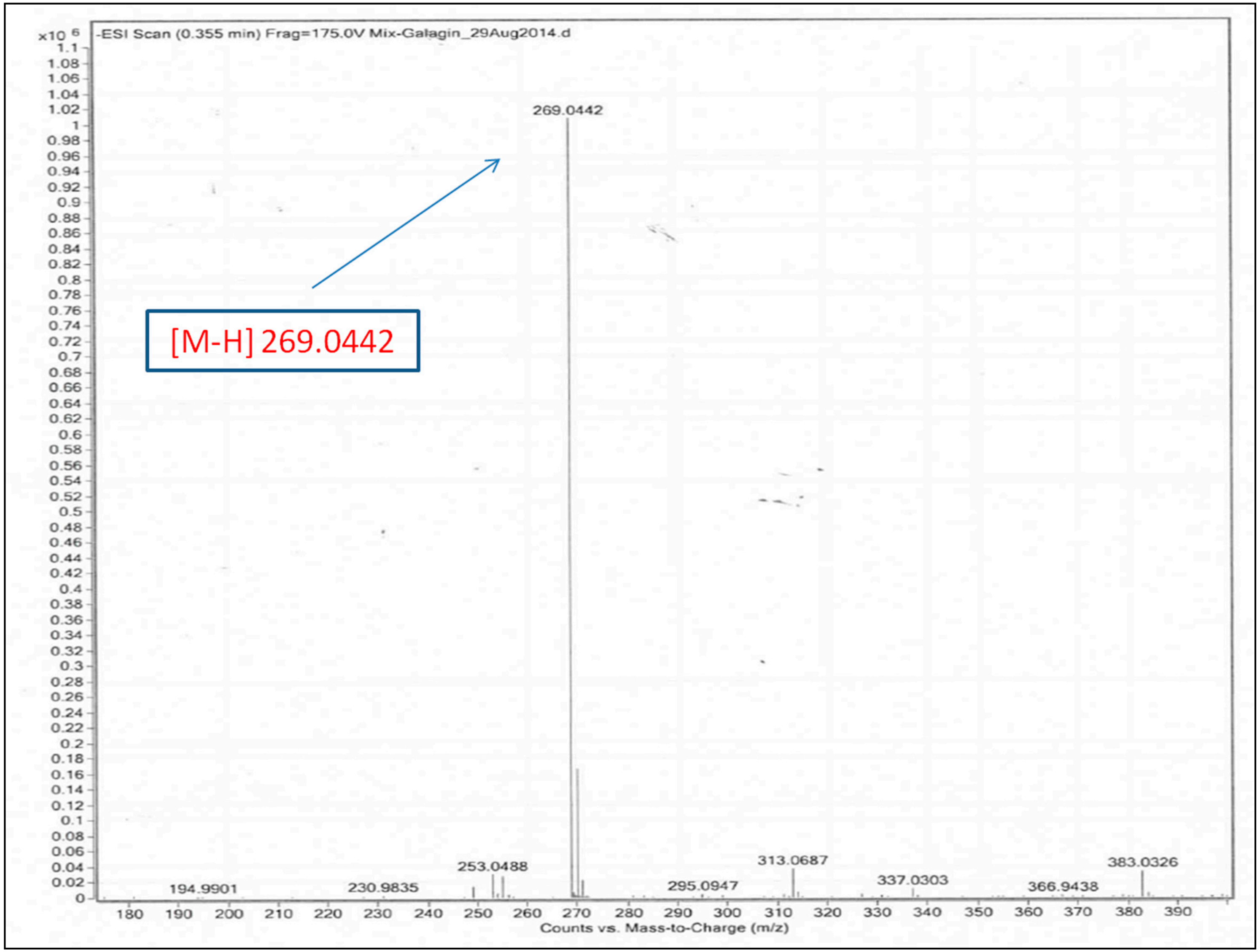

Supplement: Supplementary File 1 [file molecules-19-18936-s001.pdf]
